# Biallelic Recessive Mutations in *TLE6* and *NLRP5* Cause Female Infertility Characterized by Human Early Embryonic Arrest

**DOI:** 10.1155/2024/9278518

**Published:** 2024-06-22

**Authors:** Ruiqi Li, Mei Mei, Ling Zhou, Haijing Zhao, Min Yang, Yingshi Li, Xiaoli Chen, Wenjun Wang, Ping Yuan

**Affiliations:** ^1^ IVF Center Department of Obstetrics and Gynecology Sun Yat-sen Memorial Hospital Sun Yat-sen University, Guangzhou, China; ^2^ Guangdong Provincial Clinical Research Center for Obstetrical and Gynecological Diseases, Guangzhou, China; ^3^ IVF Center Department of Obstetrics and Gynecology The First People's Hospital of Kashgar, Kashgar, China; ^4^ IVF Center Reproductive and Genetic Hospital of Kapok, Hainan 571400, China; ^5^ State Key Laboratory of Biocontrol School of Life Sciences Sun Yat-sen University, Guangzhou, China

## Abstract

Preimplantation embryonic developmental arrest (EDA) is a common cause of unexplained female infertility. Genetic factors are believed to be one of the primary causes contributing to EDA. In this study, we identify four novel compound heterozygous mutations in *TLE6* and *NLRP5*, in two infertile female patients experiencing recurrent EDA, using whole-exome sequencing. Functional analysis revealed that the two splicing mutations in *TLE6* (c.541+2dupT) and *NLRP5* (c.2957+4A>G) resulted in aberrant RNA splicing, leading to abnormal truncations of the corresponding proteins. *In vitro* experiments further validated that a missense mutation in *NLRP5* led to increased mRNA and protein expression levels compared to wild type, when transfected into HEK293T cells. Immunofluorescence analysis confirmed the decay of the expression of TLE6 protein. Additionally, RNA sequencing results revealed significantly higher expression levels of some maternal genes in mutated embryos with *TLE6* mutations, possibly suggesting the disrupted clearance of maternal mRNA and the failure of embryo genome activation. These results highlight the role of biallelic recessive effects associated with *TLE6* and *NLRP5* variants in embryonic development, thereby widening the scope of the genetic landscape.

## 1. Introduction

More than 70 million couples worldwide suffer from infertility [1], and a significant number of them could potentially have children with the help of assisted reproductive technology (ART). However, approximately 15 to 30% of infertile couples display ovulation, tubal patency, and normal semen analyses, which leaves their infertility classified as “unexplained infertility.” [2] Factors such as oocyte maturation arrest, fertilization failure, cleavage failure, and embryonic developmental arrest (EDA) have been discovered in these patients with unexplained infertility [3–6]. EDA is characterized by low embryo quality, embryonic fragmentation, and failure to reach the blastocyst stage [7]. Genetic factors are believed to be one of the primary causes contributing to EDA.

The subcortical maternal complex (SCMC) plays a vital role in early embryonic development by facilitating zygotes to go through their initial cell divisions [8, 9]. Within human SCMC (hSCMC), multiple proteins encoded by maternal-effect genes, such as *NLRP2*, *NLRP5*, *NLRP7*, *KHDC3L*, *OOEP*, *TLE6*, and *PADI6*, are involved [10]. Among them, *TLE6* (MIM# 612399) encodes the transducin-like enhancer protein 6, a crucial member of SCMC that regulates F-actin dynamics in zygotes [8]. In mice lacking *Tle6*, embryos exhibited abnormal development, resulting in fragmented cytoplasmic vesicles [8]. *NLRP5* (MIM# 609658), the first identified mammalian maternal-effect gene, encodes an N-terminal pyrin (PYD) region, a nucleotide-binding oligomerization (NACHT) domain, and C-terminal leucine-rich repeat (LRR) domains [11, 12], which is also an indispensable component of SCMC in mice [13]. Embryos lacking *Nlrp5* in mice shows embryonic lethality at the 2-cell developmental stage [11]. Various *TLE6* and *NLRP5* variants were found to be linked to fertilization failure and EDA in human [14, 15]. However, the impact of SCMC variants on the expression of maternal-effect genes in relation to each other is still unclear.

In this study, whole-exome sequencing (WES) was performed to identify the genetic causes of infertile patients diagnosed with EDA. And we discovered four novel mutations in *TLE6* and *NLRP5*, respectively, and the related alternative splicing (AS) events caused the abnormal truncations of the corresponding proteins. *In vivo* and *in vitro* studies were also conducted to investigate the potential pathogenicity of mutations. Furthermore, transcriptome sequencing revealed significantly higher expression levels of some maternal genes in mutated embryos, which might indicate the disrupted clearance of maternal mRNA and the failure of embryo genome activation (EGA).

## 2. Materials and Methods

### 2.1. Clinical Samples

Two infertile patients who experienced multiple in vitro fertilization (IVF) and intracytoplasmic sperm injection (ICSI) failures due to EDA were recruited in this study. After obtaining ethical approval from the Ethics Committee of Sun Yat-sen Memorial Hospital of Sun-Yat Sen University and the patients' informed consent, we took the required samples. Peripheral blood samples of the probands and their family were taken for DNA and RNA extraction. For RNA sequencing (RNA-seq), the probands' and the controls' abandoned embryos from day 3 were used. Artificial oocyte activation (AOA) was performed using calcium ionophores. All retrieved oocytes were incubated in 30 *μ*L drops of A23187 (GM508 Cult-Active, Gynemed, Germany) for 15 minutes after ICSI. Subsequently, they were washed three times before being cultured according to our institution's protocol.

### 2.2. WES and Variant Filtering

Genomic DNA was extracted from peripheral blood samples. After the construction of the DNA library according to the manufacturer's instruction (Illumina, USA) and paired-end (PE) genomic sequencing on the Illumina HiSeq 4000 platform with 2 × 150 bp-long PE reads for each sample, the product was aligned to the human genome assembly GRCh37 and Single nucleotide variants (SNVs) and small indels were identified. The identified variants were annotated and classified with the ANNOVAR software. Then, the variants were prioritized based on the filtering criteria: (i) absent in the 1000 Genomes, ExAC, dbSNP, and gnomAD, or the minor allele frequency < 0.001; (ii) predicted to be pathogenic variants by SIFT, PPH-2, and Mutation Taster; (iii) genes reported related to embryonic developmental arrest; (iv) missense/splicing/frameshift/in-frame mutations were included.

### 2.3. Detection of the *TLE6* and *NLRP5* Mutations and *In Silico* Analyses

*TLE6* and *NLRP5* mutations in the probands and families were detected by performing Sanger sequencing of the polymerase chain reaction (PCR) products of the potential mutation region using specific primers (Supplementary Table 2). The mutations were named according to the Human Genome Variation Society (HGVS) standard with +1 corresponding to the A of the ATG translation initiation codon of the GenBank cDNA sequence NM_001143986.1 for *TLE6* and NM_153447.4 for *NLRP5*. The computational programs FATHMM, PolyPhen-2, and Mutation Taster were used to predict the effect of the missense variants, while PhastCons and phyloP were used for conservation analyses. Additionally, SpliceAI was conducted for the prediction of the pathogenicity of the splice variants. The Genome Aggregation Database (gnomAD) and the Exome Aggregation Consortium (ExAC) were applied to retrieve allele frequencies of the variants. And we performed the evolutionary conservation analysis of the mutated amino acid sequences with the Clustal Omega. The open state of human TLE6 and NLRP5 structure was constructed based on the AlphaFold protein structure of human TLE6 (AF-Q9H808-F1) and NLRP5 (AF-P59047-F1). The three-dimensional (3D) structural models of the proteins were constructed with ColabFold (v1.5.3) and displayed with PyMOL Molecular Graphics System (v2.5.2). Five models were generated, and the structure with the highest per-residue model confidence score (TLE6 model per-residue DeepMind development team (pLDDT) = 86.3; NLRP5 model pLDDT = 88) was selected as the probable model for analysis.

### 2.4. The Splicing Analyses of Peripheral Blood and Minigene Assay

Total RNAs extracted from the peripheral blood from proband in family 1 and the control were reverse-transcribed using Evo M-MLV Plus Reverse Transcriptase (Accurate Biology, USA) and amplified through PCR employing Q5® High-Fidelity DNA Polymerase (M0491) (New England Biolabs, USA). Simultaneously, the RNA extracted from the HEK293 cells transfected with pMini-CopGFPs (Hitrobio.tech, China) containing fragments (exon 5 to exon 9 of *TLE6* and exon 9 to exon 11 of *NLRP5*) was reverse-transcribed using HiScript II Enzyme Mix (Vazyme, China) and amplified through PCR employing 2×Taq PCR StarMix (GenStar, China). The PCR amplification products of cDNA were detected by electrophoresis, which were then further cloned, separated, and sequenced. All the primers are detailed in Supplementary Table 2.

### 2.5. The Construction of Overexpressed Vector Containing the Missense Mutations and Quantitative Real-Time PCR

The full-length coding sequence (CDS) of *NLRP5* and *TLE6* were synthesized and cloned into the pEGFP-N1 vector (Clontech, USA), including the wild-type and the variants (*TLE6*: c.1075G>A, *NLRP5*: c.1249C>T). Every vector was then confirmed by Sanger sequencing and transfected into HEK293T cells using Lipofectamine® 3000 (Invitrogen, USA). After 24 hours, the RNA extracted from the HEK293 cells was reverse-transcribed using HiScript II Enzyme Mix (Vazyme, China) and amplified through PCR employing 2×Taq PCR StarMix (GenStar, China). Furthermore, the mRNA expression levels of the target gene, GAPDH, or the ampicillin resistance gene (Ampr) on the expression plasmid were determined by a Roche LightCycler® 480 (Roche, Switzerland). The relative mRNA expression level of the target genes was calculated by the comparative Ct method. All the primers are detailed in Supplementary Table 2.

### 2.6. Western Blot Analysis

The experimental HEK293T cells were lysed in 1×RIPA lysis buffer (Beyotime, China) with protease inhibitor. Then, each protein sample was separated on a 12% SDS-PAGE and transferred onto polyvinylidene fluoride (PVDF) membranes which had been pretreated with methanol. The membranes were blocked with 5% low-fat milk powder in Tris-buffered saline (TBS) buffer for 1 hour at room temperature. After blocking, the membranes were incubated with primary antibodies overnight at 4°C. The primary antibody was the mouse anti-GFP-Tag mAb (AE012, dilution: 1 : 5000; Abclonal, China). Secondary antibody goat-anti-mouse immunoglobulin G (IgG) (#7076S, dilution:1 : 1000; Cell Signaling Technology, USA) was applied to the samples for another 1 hour at room temperature. Before incubating the antibodies, the membranes were washed with TBS-Tween-20 (0.05%) three times, each time for 10 minutes. Finally, an enhanced chemiluminescence (ECL) kit (Fdbio, China) was used for detecting the blotting signals by GelView 6000 Pro (BLT, China). The target protein expression levels were normalized to the reference protein *β*-actin by ImageJ software (ImageJ, v1.53c).

### 2.7. RNA-Seq Analysis

Four discarded embryos on day 3 from proband 1 and four donated embryos from two control patients diagnosed with fallopian tube obstruction who have conceived successfully were taken for transcriptome analysis after informed consent was obtained. A total of 8 embryo samples were tested using the DNBSEQ platform, with an average yield of 6.37 G data per sample, and the sequence length is PE100. Raw data were filtered for quality using SOAPnuke (v1.5.2). And reads containing the connector with unknown base N content greater than 5%, low-quality reads, were removed. And then, the clean reads were aligned to the human genome (GRCh38.p13) using HISAT2 (v2.0.4). Bowtie2 (v2.2.5) was used to align the clean reads to RefSeq transcripts for quantification. The expression levels of each gene were quantified using normalized transcripts per million reads (TPM) using RSEM (v1.2.12). Eventually, 16963 genes were detected. Differential expression analysis was performed using DESeq2 (vl.4.5). And we defined upregulated genes (fold change (FC) > 2 and adjusted *p* < 0.05) and downregulated genes (FC < 0.5 and adjusted *p* < 0.05) as differentially expressed genes (DEG) between the two groups. The Venn diagram plots were drawn in R (v5.12.10). Through the R package ggcor, pheatmap, enrichKEGG, and gseKEGG, the principal component analyses, Spearman's correlation coefficients, heatmap, enrichment analysis of the Kyoto Encyclopedia of Genes and Genomes (KEGG), and gene set enrichment analysis (GSEA) were all carried out in R (v5.12.10).

### 2.8. Immunofluorescent Staining

Immunofluorescent staining for oocytes and embryos from Patient 1 was modified from a previously reported protocol [8]. In brief, additional donated embryos from the patient and the control were fixed with 4% paraformaldehyde for 30 min at room temperature, washed three times with DPBS, and permeabilized in PBS containing 0.1% TritonX-100 for 10 min. Oocytes and embryos were blocked with 5% goat serum for 1 hour at room temperature (RT). Then, the oocytes and embryos were incubated with primary antibody (anti-TLE6, Abcepta, China) diluted in 1% BSA/DPBS overnight at 4°C and followed by secondary antibody for 1 h at RT after washing three times with DPBS. DNA was stained with Hoechst 33342 (Sigma, Germany). Immunofluorescent images were captured on an Olympus inverted fluorescent microscope, and the intensity of the immunofluorescent signal was analyzed with ImageJ software.

## 3. Results

### 3.1. Clinical Characteristics of the Patients

**Figure 1:**
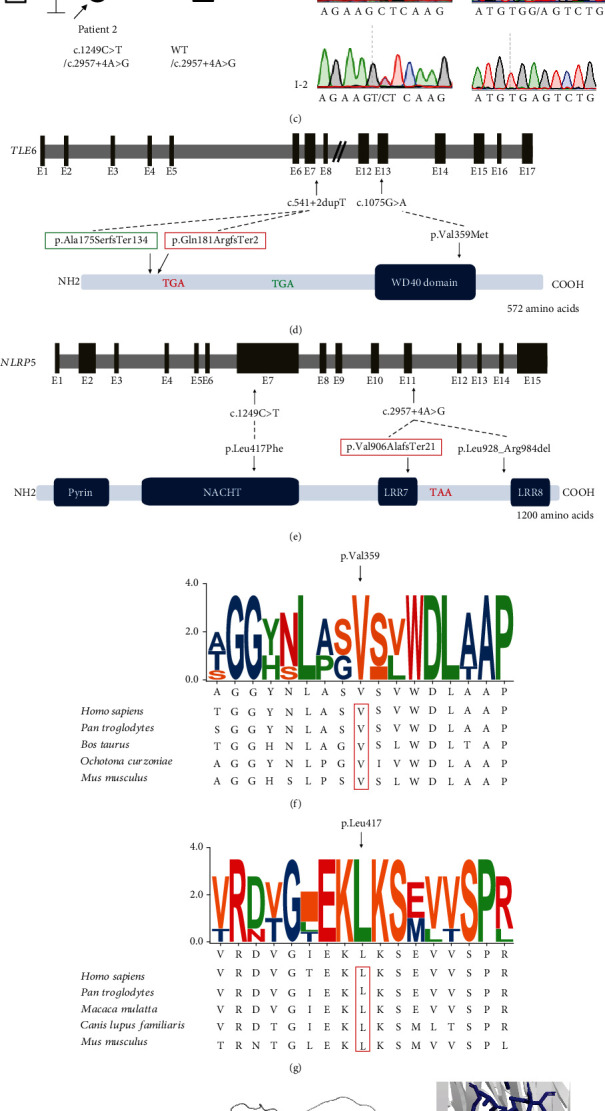
Genetic analyses of *TLE6* and *NLRP5* variants identified in the patients. (a) The phenotypes of embryos from the patients and the normal control. (b) Pedigree of the family 1 carrying *TLE6* variants. Sanger sequencing chromatograms are shown next to the pedigree. (c) Pedigree of the family 2 carrying *NLRP5* variants. Sanger sequencing chromatograms are shown next to the pedigree. (d) Locations of the *TLE6* mutations and the corresponding protein mutations. TGAs represent the PTCs. (e) Locations of the *NLRP5* mutations and the corresponding protein mutations. TAA represents the PTC. (f) Conservation analysis of the 359^th^ amino acid residue in TLE6. (g) Conservation analysis of the 417^th^ amino acid residue in NLRP5. (h) The 3D structural modeling of TLE6, WD40 domain, is shown in blue. The red sticks represent the amino acid residues at the 359th position. (i) The 3D structural modeling of NLRP5, the NACHT domain, is shown in blue, the PYD region is shown in green, and the seventh LRR domain is shown in pink. The red sticks represent the amino acid residues at the 417th position.

After the sex hormone test and the ultrasound examination, the ovarian reserve and ovulation function of the patient from family 1 who had been treated in our IVF department for primary infertility for more than 7 years were found to be normal. The couple has tried three times of IVF/ICSI attempts which all failed (Table 1). A total of 42 oocytes were collected during the three cycles, but none of the embryos reached the blastocyst stage after extended culture. All of the patient's embryos were arrested at the cleavage stage, with poor symmetry, fragmentation, and a small number of blastomeres ranging from 2 to 7 (Figure 1(a)). Despite undergoing two embryo transfer cycles with a total of four low-quality embryos, the patient was unable to achieve a viable pregnancy.

The patient from family 2 who was diagnosed with polycystic ovarian syndrome was 32 years old at the first time of examination and had experienced primary infertility for 16 years till now. She had undergone one IVF combined with late rescue ICSI and two ICSI attempts before seeking treatments in our IVF center (Table 1). A total of 29 oocytes were retrieved during the three cycles with a 44% normal fertilization rate (13/29). However, the couple failed to establish pregnancy after transferring the only one available embryo. Due to the low fertilization rate, another ART protocol including AOA was established in our center. Subsequently, the fertilization rate of two pronuclei increased to 92% (13/14). Twelve of them were cleaved leaving 4 available embryos on day 3. Unfortunately, these embryos also displayed poor symmetry and a high fragmentation rate (Figure 1(a)). After transferring one fresh and two frozen embryos, the patient was unable to achieve a successful pregnancy (Table 1).

### 3.2. Genetic and *In Silico* Analyses of the *TLE6* and *NLRP5* Variants

WES was conducted on the two patients, revealing the identification of four novel heterozygous variants. Specifically, Patient 1 exhibited the variants c.541+2dupT in intron 7 and c.1075G>A in exon 13 from *TLE6*, while Patient 2 displayed the variants c.1249C>T in exon 7 and c.2957+4A>G in intron 11 from *NLRP5* (Figures 1(b), 1(c), 1(d), and 1(e)). Sanger sequencing confirmed that both patients had compound heterozygous mutations inherited from their parents as shown in Figures 1(b) and 1(c). Furthermore, all of the variants were found to be absent or extremely rare in both gnomAD and ExAC. Computational analysis using FATHMM, PolyPhen-2, Mutation Taster, and SpliceAI predicted that both the missense variants in *TLE6* and *NLRP5* were likely to be damaging, while the splice variants were deemed pathogenic (Supplementary Table 1). Alignment of the TLE6 and NLRP5 protein showed that the amino acid residues at the 359^th^ and 417^th^ positions were highly conserved (Figures 1(f) and 1(g)).

The TLE6 protein consists of six beta-transducin repeats (residues 276-313, 326-363, 368-407, 410-447, 490-529, and 532-569), 40-amino acid motifs that often terminate in a Trp-Asp (W-D) dipeptide. The structure prediction from ColabFold suggested that mutant methionine residues within the WD40 domain resulted in alterations to the hydrophobic interactions, potentially impacting the overall stability of the protein (Figures 1(d) and 1(h)). NLRP5 features an N-terminal PYD region (residues 57-148), a NACHT domain (residues 280-602), and thirteen C-terminal LRR domains (residues 704-727, 730-753, 780-803, 809-832, 836-863, 865-892, 893-916, 950-973, 979-1002, 1007-1034, 1036-1059, 1064-1092, and 1121-1142). Similarly, it was predicted that the replacement of Leu417 by phenylalanine, located in the NACHT domain, would weaken the hydrophobic interactions, potentially destabilizing the protein's stability (Figures 1(e) and 1(i)).

### 3.3. Analysis of Alternative Splicing (AS) Events

**Figure 2:**
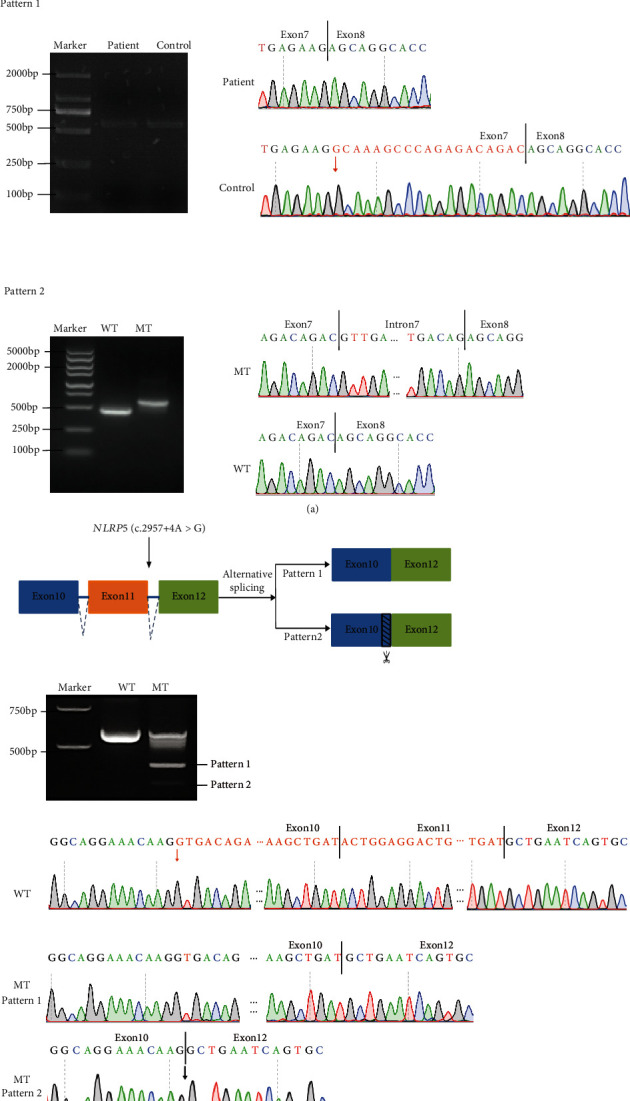
The splicing analysis related to the splicing variants in *TLE6* and *NLRP5*. (a) The two anomalous splicing patterns from the splicing mutation in *TLE6* are shown above, along with electrophoresis and Sanger sequencing chromatograms of the mutant fragments. Pattern 1 shows the 19 bp deletion of exon 7 from the RT-PCR analysis of blood samples, and pattern 2 shows the retention of 118 bp of intron 7 from the RT-PCR analysis of minigene analysis. (b) The two aberrant splicing patterns arising from the splicing mutation in *NLRP5* are depicted above, accompanied by electrophoresis results and Sanger sequencing chromatograms of the mutated fragments. Pattern 1 shows the 171-bp deletion of exon 11, and pattern 2 shows the deletion of 242 bp including the entire exon 11 and partial exon 10.

To identify and verify the AS events connected to the two novel splicing variants in *TLE6* and *NLRP5*, both *in vitro* and *in vivo* investigations were carried out. Interestingly, the prediction results from RNA Splicer showing two alternative splicing outcomes associated with the c.541+2dupT variant in *TLE6* were confirmed*. In vivo*, RT-PCR analysis of blood samples revealed an abnormal splicing pattern, classified as pattern 1, resulting in the deletion of 19 base pairs (bp) within exon 7. On the other hand, *in vitro* minigene analysis showed that the c.541+2dupT variant in *TLE6* led to the retention of the entire 118 bp intron 7, classified as pattern 2 (Figure 2(a)). Pattern 1 introduced a premature stop codon at position 308, leading to the truncation of 265 amino acids at the C terminus of the TLE6 protein (p.Ala175SerfsTer134). Conversely, pattern 2 introduced a premature stop codon at position 182, resulting in the truncation of 391 amino acids (p.Gln181ArgfsTer2) (Figure 1(d)).

Similarly, *in vitro* minigene analysis showed that the c.2957+4A>G variant in *NLRP5* leads to the deletion of the entire 171 bp exon 11, classified as pattern 1, and the deletion of 242 bp including the entire exon 11 and partial exon 10, classified as pattern 2. Pattern 1 caused the truncation of 57 amino acids at the C terminus of the NLRP5 protein. And pattern 2 introduced a premature stop codon at position 926, resulting in the truncation of 275 amino acids (p.Val906AlafsTer21) (Figure 1(e)).

### 3.4. Expression of the Missense Mutations in *TLE6* and *NLRP5* in HEK 293T Cells and the Arrested Embryos

**Figure 3:**
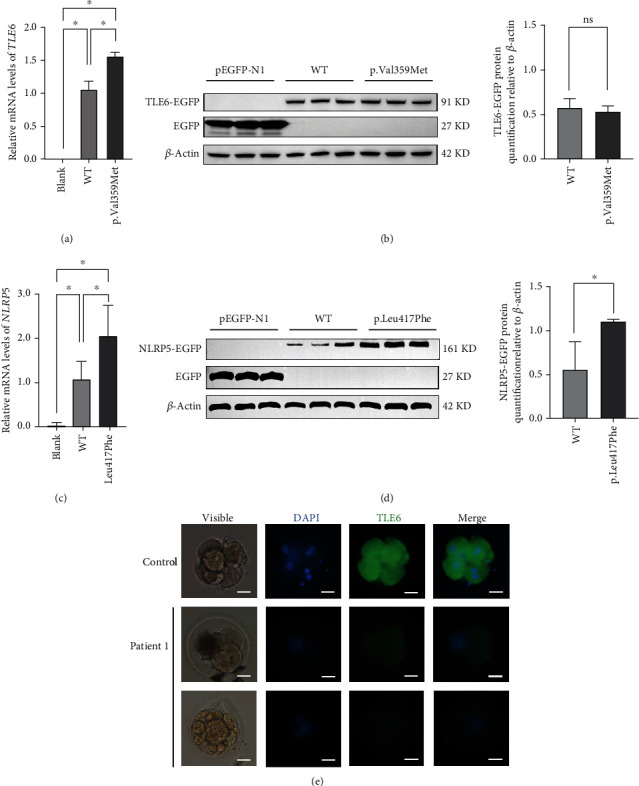
Expression of the missense mutations in *TLE6* and *NLRP5* in HEK 293T cells and immunofluorescence analysis of arrested embryos in Patient 1. (a) The difference in *TLE6* gene expression levels between the wild and mutant type as assessed by qPCR. (b) Quantification of TLE6-EGFP fusion protein expression levels based on the intensity of bands of western blot. (c) The difference in *NLRP5* gene expression levels between the wild and mutant type as assessed by qPCR. (d) Quantification of NLRP5-EGFP fusion protein expression levels based on the intensity of bands of western blot.^∗^*p* value < 0.05; ns: nonsignificant. (e) The immunofluorescence staining of embryos on day 3 of Patient 1 that had been immunolabeled with antibodies against TLE6 (green) and stained with DAPI (blue) to see the DNA. Scale bar = 50 *μ*m.

Both wild-type and mutant vectors were transfected into HEK 293T cells to evaluate the expression of the identified missense mutations in *TLE6* and *NLRP5*. The relative levels of mutant *TLE6* mRNA in HEK 293T cells were higher than that in the wild type (*p* < 0.05) (Figure 3(a)). Nevertheless, the western blot analysis showed that there was no significant difference in TLE6 protein level between the two groups (Figure 3(b)). Furthermore, the relative levels of mutant *NLRP5* mRNA and protein were higher than those of the wild type in HEK 293T cells (*p* < 0.05) (Figures 3(c) and 3(d)). Moreover, immunofluorescence analysis demonstrated obviously weaker TLE6 signals in the embryos from the proband compared to the control group (Figure 3(e)).

### 3.5. RNA-Seq Analysis of the Arrested Embryos

**Figure 4:**
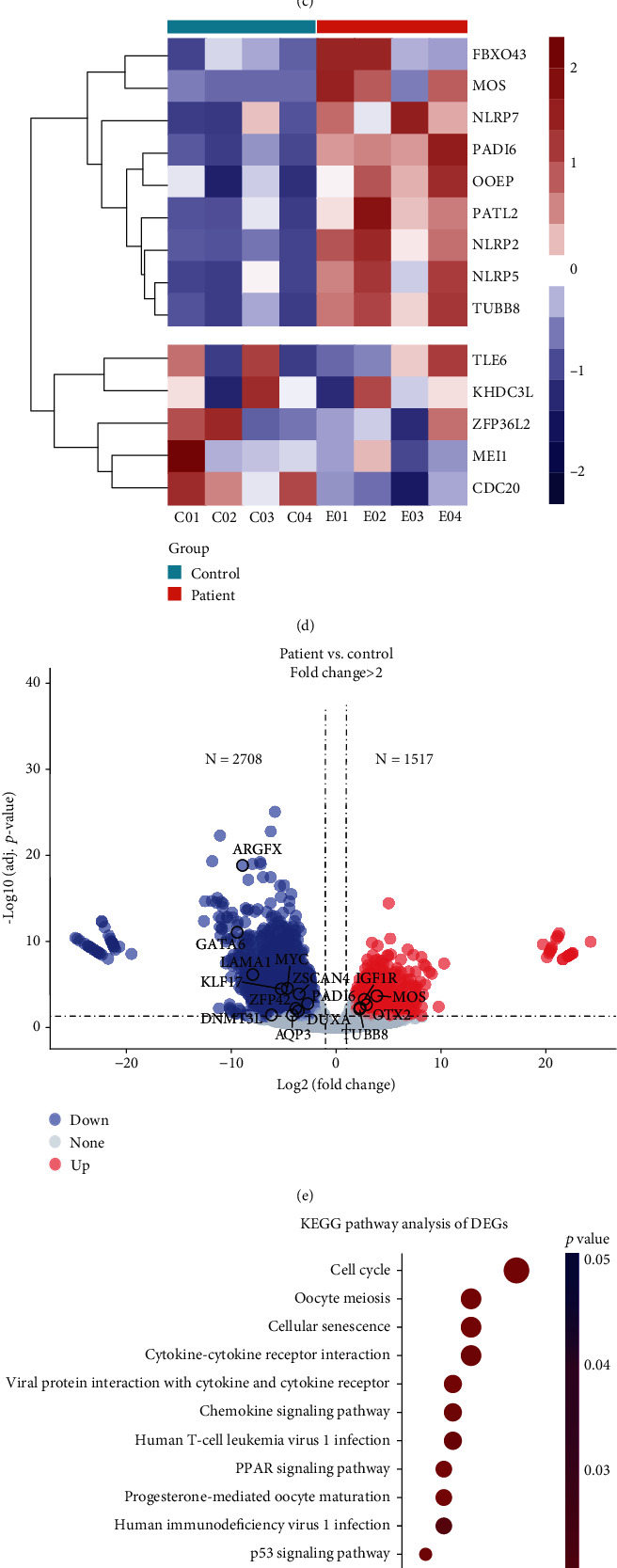
RNA-seq analysis of the arrested embryos of Patient 1. (a) The Venn diagram displays how many overlapping and unique genes are expressed in the two groups. (b) PCA analysis shows the distribution of the embryo samples between the group patient and the group control. (c) The Pearson correlation analysis of RNA-seq results. ^∗∗∗^*p* value < 0.001. (d) The heat map shows the expression of genes related to SCMC and embryo development. (e) Volcano plot highlights DEGs (upregulated in red, downregulated in blue, and insignificant in pale gray; circled dots are embryo development related). (f) The cluster diagram shows the KEGG pathway analysis of the DEGs. (g) The gene set enrichment analysis of the DEGs.

To investigate the potential effects of *TLE6* variants on its own expression and that of other maternal-effect genes, RNA-seq was performed on the day 3 embryos from the proband and the control patients. As group control was characterized by 1371 uniquely expressed genes, we evaluated the gene expression levels of 4 samples in group patients and discovered that 14383 genes were expressed in all the samples (Figure 4(a)). Distinct clusters of gene expression were observed in both the patient and control groups by principal component analysis (PCA) (Figure 4(b)). The Pearson correlation coefficient analysis revealed significant transcriptome differences between the patient and control embryos (Figure 4(c)). Moreover, we found that the expressions of several known EDA-related genes were clustered and highly upregulated in group patients, including *FBXO43*, *MOS*, *NLRP2*, *NLRP5*, *NLRP7*, *OOEP*, *PADI6*, *PATL2*, and *TUBB8*, while the expressions of other 5 genes were clustered including hSCMC genes (*TLE6* and *KHDC3L*) (Figure 4(d)). DEG analyses, however, indicated that the expressions of some genes involved in EGA were significantly downregulated in the patient group, while the expressions of several maternal genes were found to be abundant (Figure 4(e)). Moreover, KEGG enrichment analysis revealed that the DEGs were predominantly enriched in pathways associated with cell cycle, oocyte meiosis, and cellular senescence (Figure 4(f)). GSEA also showed that the gene set linked to the cell cycle was significantly upregulated in the patient group (Figure 4(g)).

## 4. Discussion

In this study, we successfully identified four novel mutations in *TLE6* and *NLRP5* in infertile female patients diagnosed with EDA, both of which encode the SCMC complex. By utilizing RT-PCR and minigene assays, we verified that splicing mutations in *TLE6* (c.541+2dupT) and *NLRP5* (c.2957+4A>G) induce AS events, ultimately causing the abnormal truncations of the corresponding proteins. Additionally, RNA-seq analysis of arrested embryos revealed that molecular changes in *TLE6* may contribute to the probable impaired clearance of maternal mRNA in these embryos.

Recent evidence strongly suggests that the SCMC plays a vital role in the initial wave of transcript clearance driven by maternal factors [16], which is necessary for the normal development of embryos. The SCMC is composed of multiple proteins, with TLE6 and NLRP5 playing a crucial role in maintaining the structural integrity of the SCMC. Multiple studies have linked mutations in SCMC to fertilization failure, EDA, and recurrent miscarriages [16]. In this study, the probands from the two families failed to conceive undergoing several cycles of IVF/ICSI, for the majority of the patients' embryos were arrested at the cleavage stage. Additionally, the number of cells in blastomeres was relatively low, with poor symmetry and severe fragmentation, which was in accordance with the EDA phenotype described in previous studies [7, 16].

Mu et al. identified two compound heterozygous missense variants in *NLRP5* (c.1598G>C and 1919 T>G) which caused the incidence of total fertilization failure, although they performed ICSI and AOA [17]. However, other studies showed that the oocytes with the mutation of *NLRP5* could be fertilized by IVF and ICSI [18]. Similarly, in our study, Patient 2 with the compound heterozygous mutations of *NLRP5* (c.1249C>Tand c.2957+4A>G) experienced low fertilization in the multiple cycles. Interestingly, the fertilization rate seemingly could be improved by ICSI combined with AOA. Even so, due to maternal gene mutations, AOA could not improve the embryo qualities.

*In vitro* and *in vivo* investigations verified that the splicing mutations in *TLE6* resulted in two distinct splicing patterns: retained intron and alternative 5′ splice site (A5SS). Since NLRP5 was found to be barely expressed in peripheral blood lymphocytes, we conducted *in vitro* minigene analysis, which indicated that the splicing variant in NLRP5 would result in exon skipping and alternative 5′ splice site (A5SS). These alterations led to the generation of premature termination codons (PTCs), which are likely to trigger nonsense-mediated mRNA decay (NMD) [19]. Based on these findings, it was reasonable to speculate that the aberrant splicing patterns would result in NMD, causing the degradation of the nascent peptides, consistent with our observations from immunofluorescence analysis in the arrested embryos with *TLE6* mutations.

Previous studies have demonstrated that EGA usually begins on day 3 in human 8-cell embryos, accompanied by significant degradation of maternal mRNA and defects which might be linked to early developmental arrest [20]. Our DEG analysis of RNA-seq uncovered the higher expressions of some maternal genes such as *TUBB8*, *MOS*, *IGF1R*, *PADI6*, and *OTX2* in *TLE6* mutated embryos. We also found that several hSCMC genes such as *NLRP2*, *NLRP5*, *NLRP7*, and *OOEP* were clustered and relatively upregulated in the arrested embryos, although the differences were not significant. In addition, the KEGG and GSEA analysis revealed a notable association between DEGs involved in regulating the cell cycle. These findings provide further insight into the probable impaired clearance of maternal mRNA affected by errors in *TLE6* within arrested embryos. Furthermore, Tong et al. have found that the expression of several genes that should have been significantly increased in 8-cell stage embryos is significantly downregulated in *OOEP* mutant embryos, suggesting the mutation modestly affected ZGA in human embryos [18]. Similarly, we also noticed the downregulation of EGA-related genes like *DUXA*, *ZSCAN4*, *GATA6*, *KLF17*, and *MYC* in *TLE6* mutant embryos, probably suggesting the affected ZGA in arrested embryos. *In vitro* experiments on the missense mutations in *TLE6* and *NLRP5* revealed that the expressions of mutated mRNA were higher than that of the wild-type, potentially worsening the deficiency in maternal mRNA clearance.

Our immunofluorescence analysis demonstrated obviously weaker TLE6 signals in the arrested embryos compared to the control group. This suggests that the health status of these embryos, which may include conditions such as developmental arrest or degeneration, could affect protein expression levels, potentially impacting the reliability of our results. We recognize that employing an antibody targeting a housekeeping gene, renowned for its consistent expression under various conditions, would have served as an optimal control to confirm the specificity of the observed TLE6 downregulation. The absence of such a control is a limitation we acknowledge. Additionally, we are cognizant that our RNA-seq analysis is conducted on a relatively small sample size of embryos, which might restrict the robustness and applicability of our findings. We have noted this limitation and recommend a cautious interpretation of our data, underscoring the necessity for future studies involving larger sample sizes and appropriate controls to corroborate our findings.

In summary, we have identified novel biallelic mutations in *TLE6* and *NLRP5* in infertile female patients with EDA. Through *in vitro* and *in vivo* experiments, we have confirmed the AS events related to the splice variants, which lead to the generation of PTCs. As a result of these changes, the corresponding protein is impaired, leading to the likely compromised clearance of maternal RNAs, which was confirmed *in vitro* experiments, and subsequently impacting embryonic development. These findings not only broaden the genetic and phenotypic spectrum of *TLE6* and *NLRP5* variants but also contribute to a valuable understanding of the mRNA clearance defects in arrested embryos.

## 5. Web Resources

1. BWA aligner (0.5.9-r16) software (http://bio-bwa.sourceforge.net/)
2. SAM tool (http://samtools.sourceforge.net/)
3. 1000 Genomes (http://www.1000genomes.org/)
4. ExAC (http://exac.broadinstitute.org/)
5. dbSNP (https://www.ncbi.nlm.nih.gov/snp/)
6. GnomAD (https://gnomad.broadinstitute.org)
7. FATHMM (http://fathmm.biocompute.org.uk/fathmm-xf/index.html)
8. PPH-2 (http://genetics.bwh.harvard.edu/pph2/index.shtml)
9. Mutation Taster (http://www.mutationtaster.org/)
10. PhastCons PhyloP ((http://www.mutationtaster.org/)
11. SpliceAI (https://github.com/Illumina/SpliceAI)
12. RNA Splicer (https://rddc.tsinghua-gd.org/search-middle?to=SplitToolModel)
13. Clustal Omega(http://www.clustal.org)
14. AlphaFold Protein Structure Database (https://alphafold.ebi.ac.uk/)
15. ColabFold (https://colab.research.google.com/github/sokrypton/ColabFold/blob/main/AlphaFold2.ipynb)
16. PyMOL (https://www.pymol.org/pymol.html)
17. SOAPnuke software (https://github.com/BGI-flexlab/SOAPnuke)
18. HISAT2 (http://www.ccb.jhu.edu/software/hisat)
19. Bowtie2 (http://bowtiebio.sourceforge.net/%20Bowtie2%20/index.shtml)
20. RSEM (https://github.com/deweylab/RSEM)
21. DESeq2 (http://www.bioconductor.org/packages/release/bioc/html/DESeq2.html)
22. Venn diagram (http://en.wikipedia.org/wiki/Venn diagram)
23. PCA (https://github.com/kevinblighe/PCAtools)
24. Spearman correlation analysis (http://127.0.0.1:24558/help/library/ggcor/help/ggcor)
25. pheatmap package (https://cran.rproject.org web/packagespheatmap/index.html)
26. enrichKEGG (https://rdrr.io/github/GuangchuangYu/clusterProfiler/src/R/enrichKEGG.R)
27. gseKEGG (https://rdrr.io/github/GuangchuangYu/clusterProfiler/man/gseKEGG.html),
28. HGVS (http://www.hgvs.org/mutnomen/)

## Figures and Tables

**Table 1:** The clinical characteristics of the patients in IVF and ICSI cycles.

Patient	Cycle	Age (years)	IVF/ICSI attempts	Total oocytes retrieved	MII oocytes	Normally fertilized embryos	Available embryos of day 3	Blastocyst formation	Outcomes
Patient 1 in Family 1	1	33	Half-ICSI	10	9	8	7	0	Five available embryos for extended culture were all arrested on day 3, and two for transfer failed to yield a successful pregnancy.
	2	33	IVF	12	10	5	4	0	Two available embryos for extended culture were arrested on day 3, and two for transfer failed to yield a successful pregnancy.
	3	34	IVF	20	16	13	7	0	All embryos were arrested during blastocyst culture.

Patient 2 in Family 2	1	32	IVF + late r-ICSI	15	12	1 (IVF) + 9 (late r-ICSI)	1	NA	A single viable fresh embryo was transferred but failed to establish a pregnancy.
	2	33	ICSI	13	9	2	0	NA	No available embryos for transfer.
	3	33	ICSI	1	1	1	0	NA	No available embryos for transfer.
	4	34	ICSI + AOA	14	14	13	4	NA	One grade II 9-cell embryo was transferred but failed to establish a pregnancy. The remaining three embryos were cryopreserved.
	5	35	FET	NA	NA	NA	3	0	All available embryos were arrested during blastocyst culture. Transferred were one grade II 11-cell and one grade II 8-cell embryo on day 4, but both failed to establish a pregnancy.

Abbreviations: IVF: *in vitro* fertilization; ICSI: intracytoplasmic sperm injection; r-ICSI: rescue intracytoplasmic sperm injection; NA: not available; AOA: artificial oocyte activation; FET: frozen-thawed embryo transfer.

## Data Availability

The datasets used and/or analyzed during the current study are available from the corresponding author on reasonable request.

## Supplementary Materials

Supplementary 1 Supplemental Table 1: the characteristic and bioinformatic predictions of the novel variants in *TLE6* and *NLRP5* identified in our study.

Supplementary 2 Supplemental Table 2: list of primers used in this study.
