# Supplementary material for: Biallelic Recessive Mutations in TLE6 and NLRP5 Cause Female Infertility Characterized by Human Early Embryonic Arrest
Source: Hum Mutat. 2024 Jun 22;2024:9278518. doi: 10.1155/2024/9278518 (PMC11919057; doi:10.1155/2024/9278518)
Supplement: Supplementary 1 — Supplemental Table 1: the characteristic and bioinformatic predictions of the novel variants in TLE6 and NLRP5 identified in our study. [file 9278518.f1.docx]

**Supplementary Table 1. The characteristic and bioinformatic predictions of the novel variants in *TLE6* and *NLRP5* identified in our study**

| **Gene** | **Position on chromosome 19** | **cDNA**  **change** | **Protein change** | **Mutation type** | **GnomAD** | **ExAC** | **Prediction for**  **conservation^a^** | | **Mutation**  **assessment^b^** | | | | **ACMG classification** |
| --- | --- | --- | --- | --- | --- | --- | --- | --- | --- | --- | --- | --- | --- |
|  |  |  |  |  |  |  | PhastCons | PhyloP | FATHMM | PPH2 | Mutation Taster | Splice  AI |  |
| *TLE6* | 2987237 | c.541+2dupT | p.Ala175SerfsTer132  p.Gln181ArgfsTer2 | Splice  variant | NA | NA | 0.01 | 2.52 | NA | NA | N | Pathogenic | Pathogenic |
|  | 2989614 | c.1075G>A | p.Val359Met | Missense | 7.99×10^-6^ | 1.68×10^-5^ | 1.00 | 2.38 | T | P | D | NA | Likely Pathogenic |
| *NLRP5* | 56538848 | c.1249C＞T | p.Leu417Phe | Missense | 3.18×10^-5^ | 1.68×10^-5^ | 0.03 | 0.98 | D | P | N | NA | Likely Pathogenic |
|  | 56552462 | c.2957+4A＞G | p.Val906AlafsTer21  p.Leu928_Arg984del | Splice  variant | NA | NA | 1.00 | 2.79 | NA | NA | D | Pathogenic | Pathogenic |

Abbreviation: GnomAD, **The Genome Aggregation Database**; dbSNP: The Single Nucleotide Polymorphism Database; ExAC, the Exome Aggregation Consortium; NA,not available; FATHMM, Functional Analysis Through Hidden Markov Models; PPH2, PolyPhen-2; ACMG, The American College of Medical Genetics and Genomics; T:Tolerated, D: Deleterious in FATHMM programs; P: Possibly damaging in Polyphen programs; D: Disease causing, N: Polymorphism in Mutation Taster programs.

^a^Prediction for conservation by PhastCons and PhyloP.

^b^Mutation assessment by FATHMM, PPH2, Mutation Taster and SpliceAI.
