# Supplementary material for: Biallelic Recessive Mutations in TLE6 and NLRP5 Cause Female Infertility Characterized by Human Early Embryonic Arrest
Source: Hum Mutat. 2024 Jun 22;2024:9278518. doi: 10.1155/2024/9278518 (PMC11919057; doi:10.1155/2024/9278518)
Supplement: Supplementary 2 — Supplemental Table 2: list of primers used in this study. [file 9278518.f2.docx]

**Supplementary table 2. List of primers used in this study**

| Target gene | Variants | Species | Sequences (5’-3’) |
| --- | --- | --- | --- |
| PCR primers for validation of variants in peripheral blood samples | | | |
| *TLE6* | c.541+2dupT | Human | F:TGAGGCAGGAGAACCACTTGAA |
|  |  | Human | R:TGTTAGGACCAGAAGACCTCGGA |
| *TLE6* | c.1075G>A | Human | F:TGGCAAAGGGACAATACTTGA |
|  |  | Human | R:TTGGCTGGGAGAGTGCTATATC |
| *NLRP5* | c.1249C>T | Human | F:TGTTGTTCATCATTGACGGTTT |
|  |  | Human | R:ACAACCGTGAGCTGCTCG |
| *NLRP5* | c.2957+4A>G | Human | F:ACTATGGGGGTGATACGTCTTT |
|  |  | Human | R:ACAGTCAGTTCTCATAATTGGAGG |
| PCR primers for validation of variants in vectors | | | |
| *TLE6* | c.541+2dupT | Human | F:GATATACACTGTTTGAGATGAGGA |
|  |  | Human | R:TGCCACTACACAGACTGATT |
| *TLE6* | c.1075G>A | Human | F:CGCAAATGGGCGGTAGGCGTG |
|  |  | Human | R:TGTGGGACAGGCTCATTATC |
| *NLRP5* | c.1249C>T | Human | F:CAAGGTGGACTCTACCAGGGAA |
|  |  | Human | R:GAAGGATGTTCATGTGAARACAGAGC |
| *NLRP5* | c.2957+4A>G | Human | F:GATATACACTGTTTGAGATGAGGA |
|  |  | Human | R:GTGACTAGAGGTTCACAGGA |
| RT-PCR primiers of peripheral blood samples | | | |
| *TLE6* | c.541+2dupT | Human | F:GGCAGAACATCACAAGCAGATAGG |
|  |  | Human | R:CTCTTCCATGCATCTTCAAAGTCC |
| RT-PCR primiers of minigene analysis | | | |
| *TLE6* | c.541+2dupT | Human | F:GGCTAACTAGAGAACCCACTGCTTA |
|  |  | Human | R:GTGCCTTGCTTTCCTGCCCCAGGCCTGGTGCCTGCT |
| *NLRP5* | c.2957+4A＞G | Human | F:GGCTAACTAGAGAACCCACTGCTTA |
|  |  | Human | R:TCCAGGTCCTGGAGATGACAAGAT |
| qPCR primiers | | | |
| *TLE6* | c.1075G>A | Human | F:GCCCTCCCTGCATGTGAA |
|  |  | Human | R:CCAGATGTTGTAGCCCTTGA |
| *NLRP5* | c.1249C>T | Human | F:GCTCACCTTTTCCAGCTACGG |
|  |  | Human | R:ATGCTCCATAATACTCATGCAAG |
| β-actin | —— | Human | F:CCTGGCACCCAGCACAAT |
|  |  | Human | R:GGGCCGGACTCGTCATAC |

Abbreviation: PCR, polymerase chain reaction; RT-PCR, reverse transcription-polymerase chain reaction; qPCR, quantitative real-time polymerase chain reaction.
